# Eye behavior predicts susceptibility to visual distraction during internally directed cognition

**DOI:** 10.3758/s13414-020-02068-1

**Published:** 2020-06-04

**Authors:** Sonja Annerer-Walcher, Christof Körner, Roger E. Beaty, Mathias Benedek

**Affiliations:** 1 University of Graz, Universitätsplatz 2, 8010 Graz, Austria; 2 Pennsylvania State University, University Park, PA USA

**Keywords:** Eye tracking, Visual distraction, Internally directed cognition, Divergent thinking, Creativity

## Abstract

When we engage in internally directed cognition (e.g., planning or imagination), our eye behavior decouples from external stimuli and couples to internal representations (e.g., internal visualizations of ideas). Here, we investigated whether eye behavior predicts the susceptibility to visual distraction during internally directed cognition. To this end, participants performed a divergent thinking task, which required internally directed attention, and we measured distraction in terms of attention capture by unrelated images. We used multilevel mixed models to predict visual distraction by eye behavior right before distractor onset. In Study 1 (*N* = 38), visual distraction was predicted by increased saccade and blink rate, and higher pupil dilation. We replicated these findings in Study 2 using the same task, but with less predictable distractor onsets and a larger sample (*N* = 144). We also explored whether individual differences in susceptibility to visual distraction were related to cognitive ability and task performance. Taken together, variation in eye behavior was found to be a consistent predictor of visual distraction during internally directed cognition. This highlights the relevance of eye parameters as objective indicators of internal versus external attentional focus and distractibility during complex mental tasks.

**Electronic supplementary material:**

The online version of this article (10.3758/s13414-020-02068-1) contains supplementary material, which is available to authorized users.

During activities like planning and imagination, attention is mainly focused on the processing of internally generated information, thus constituting internally directed cognition (IDC); in contrast, activities like reading or searching constantly rely on the processing of sensory information and thus are viewed as externally directed cognition (EDC; Andrews-Hanna, Smallwood, & Spreng, 2014; Chun, Golomb, & Turk-Browne, 2011; Dixon, Fox, & Christoff, 2014). Because attentional resources are limited, pursuing extended IDC or EDC activities requires one to maintain a sustained attentional focus. However, sometimes we get distracted by irrelevant internal or external information (Smallwood & Schooler, 2015). For example, while reading, we might start thinking about a worrying event (mind wandering). And while planning a lecture, we might get distracted by a notification from our email program (visual distraction). It can be assumed that the level of absorption and distractibility varies over time, and previous research showed that eye behavior predicts mind wandering (i.e., internal distraction during EDC; Smallwood et al., 2011). Here, we tested whether eye behavior also reflects the variability of absorption during IDC and thus predicts susceptibility to external visual distraction.

Early work in this field observed first evidence that the eyes exhibit particular behaviors during internal tasks (Singer, Greenberg, & Antrobus, 1971). Daydreaming was found to lead to “ocular quiescence,” while active thinking (i.e., visual imagery and suppression of a wish) was associated with considerably more eye movements and blinks. Recent studies extended this research by comparing internal to external tasks and conditions. One study compared divergent thinking and letter-reading, two tasks that differ in their focus of attention (IDC vs. EDC), but do not require eye movements (Walcher, Körner, & Benedek, 2017). IDC was associated with more and longer blinks, and fewer microsaccades, more saccades, higher microsaccade amplitude, and larger pupil diameter than EDC. Another study varied the focus of attention within tasks by means of conditional stimulus masking and found that IDC was again associated with more blinks and larger pupil diameter, but fewer saccades compared with EDC (Benedek, Stoiser, Walcher, & Körner, 2017b). Neuroscientific research further revealed that IDC and EDC also differ in brain activation: IDC consistently involves increased EEG alpha activity in posterior brain regions (Benedek, Bergner, Könen, Fink, & Neubauer, 2011; Benedek, Schickel, Jauk, Fink, & Neubauer, 2014b) as well as reduced occipital brain activation (Benedek, Stoiser, et al., 2017b), brain patterns that are indicative of reduced sensory processing (for reviews, see Benedek, 2018; Fink & Benedek, 2014).

What are the reasons for differences in eye behavior between IDC and EDC? Changes of eye behavior during IDC are thought to reflect perceptual decoupling from sensory information and general visual disengagement (Smallwood & Schooler, 2006). Moreover, during IDC, eye behavior may get coupled to internal processes (Bone et al., 2018; Ferreira, Apel, & Henderson, 2008; Johansson & Johansson, 2013) and characteristics of imagined stimuli (e.g., luminance, size, distance; Brandt & Stark, 1997; Laeng & Sulutvedt, 2014; Sulutvedt, Mannix, & Laeng, 2018). The specific differences in eye behavior between IDC and EDC may further depend on actual characteristics of IDC and EDC activities: When the external task or condition requires constant eye movements (e.g., reading), eye activity during EDC is typically higher than during IDC (Benedek, Stoiser, et al., 2017b). In contrast, when the external task requires focusing on a fixed target, eye activity during IDC is typically higher (Annerer-Walcher, Körner, & Benedek, 2018; Franklin, Broadway, Mrazek, Smallwood, & Schooler, 2013a; Walcher et al., 2017).

Recent research has increasingly studied internal attention in the context of creative cognition (Benedek, 2018; Salvi & Bowden, 2016). Creative thinking relies on imagination and the recombination of memory elements, but does not typically require constant sensory input and therefore can be viewed as prime example of IDC (Benedek & Fink, 2019). For example, in creative idea-generation tasks (i.e., divergent thinking tasks), people are required to find novel, effective solutions to open-ended problems (e.g., alternate uses for a brick). Such tasks keep people engaged in imagination for several minutes, which enables the study of extended periods of goal-directed IDC. This represents an important complement to research that has often focused on very short tasks (e.g., mental arithmetic) or spontaneous forms of IDC (i.e., mind wandering). Indeed, creative thinking was found to be associated with eye behaviors reflecting visual disengagement as evidenced by increased blink count and blink duration, and by reduced microsaccade activity (Walcher et al., 2017). Further studies suggest that turning attention inward may even play a functional role for creativity in terms of increasing creative performance. Right before people have “Aha”-experiences in creative problem-solving tasks, EEG alpha activity was found to be increased (Kounios & Beeman, 2014), and people blinked longer and looked away from the problem more often (Salvi, Bricolo, Franconeri, Kounios, & Beeman, 2015). Similarly, creative idea generation is generally associated with increased posterior EEG alpha activity, and alpha activity is even higher right before people get more creative ideas (Fink & Benedek, 2014). Taken together, creative cognition manifests a clear neurophysiological pattern oriented to avoid visual distraction and to suppress the processing of irrelevant sensory input, serving to shield ongoing internal processing such as imagination.

As IDC and EDC differ in eye behavior, these differences can be used to identify shifts of focus between internal and external attention. First, relevant evidence comes from research on mind-wandering research, which represents spontaneous episodes of internal attention focus during EDC. Mind-wandering episodes were found to be characterized by larger pupil diameter, more blinks, and longer fixations (Franklin, Broadway, et al., 2013a; Konishi, Brown, Battaglini, & Smallwood, 2017; Reichle, Reineberg, & Schooler, 2010; Smallwood et al., 2011; Smilek, Carriere, & Cheyne, 2010; Unsworth & Robison, 2018). Here, we ask the question whether eye behavior is also indicative of shifts of attention during IDC—that is, when attention is spontaneously drawn away from an internal task towards the external environment. Hence, does eye behavior predict the susceptibility to visual distraction during internally directed cognition?

## Present study

Maintaining sustained attention for task-related information while ignoring distraction is cognitively demanding, especially over longer periods. Depending on task characteristics and cognitive resources, attentional focus may vary from deep absorption to considerable susceptibility to distraction. Because internally and externally directed attention differ in eye behavior, we explored the question of whether eye behavior also predicts variability in the susceptibility to visual distraction during an internal task. In a previous study, we found that during short, highly demanding IDC (i.e., mental arithmetic), attentional focus was very high, as indicated by very low susceptibility to visual distraction (Annerer-Walcher et al., 2018). In the present work, we examined longer lasting IDC, which is expected to exhibit higher variability in task focus and thus in susceptibility to distraction. Specifically, we had participants work on a creative idea-generation task while continuously probing their current susceptibility to visual distraction with the presentation of irrelevant distractor pictures. We investigated whether eye behavior right before distractor onset predicts the likelihood of visual distraction and thus represents a graded index of attentional focus. We considered four eye parameters (pupil diameter, fixation disparity, blink rate, and saccade rate) that were sensitive to attentional focus in previous studies. It is still unclear how susceptibility to visual distraction in the idea-generation task is reflected in eye behavior. If high susceptibility to visual distraction represents a gradual shift towards external attention focus, we would expect that visual distraction can be predicted by an eye behavior pattern more compatible with EDC: smaller pupil diameter, smaller fixation disparity, fewer blinks, and fewer saccades (Benedek, Stoiser, et al., 2017b; Salvi et al., 2015; Walcher et al., 2017). In contrast, if high susceptibility to visual distraction reflects reduced task focus (e.g., due to executive failure in maintaining internal attentional focus), we would expect that visual distraction is predicted by an eye behavior pattern that is more typical for mind wandering: larger pupil diameter, more blinks, and more saccades (Franklin, Broadway, et al., 2013a; Konishi et al., 2017; Reichle et al., 2010; Smallwood et al., 2011; Smilek et al., 2010; Unsworth & Robison, 2018).

## Study 1

### Method

#### Participants

Thirty-eight adults (26 female), ages 19 to 47 years (*M* = 25.74, *SD* = 6.07), participated in Study 1 for payment (€10/h). Most participants were students of local universities (84%). Twenty-five participants had normal vision, and 13 participants had corrected-to-normal vision (soft contact lenses) and reported no strabismus or other medical conditions affecting vision. Three additional participants were excluded from analyses because of excessive missing data (>50%) due to eye-tracker malfunction. All participants gave written informed consent. The study procedure was approved by the local ethics committee.

Sample size was determined a priori based on previous studies examining eye behavior during IDC and EDC (Annerer-Walcher et al., 2018; Benedek, Stoiser, et al., 2017b; Smallwood et al., 2011; Unsworth & Robison, 2016) and suggestions for generalized linear mixed effect models (Brysbaert & Stevens, 2018). With the final sample of 38 participants and 80 distractor pictures per participant, a total of 3,040 trials were realized, from which 2,219 valid trials were subjected to further analysis (for further details on data exclusion, see Data Processing section). Post hoc power simulations using the *powerSim* function of the *simr* package (Green & MacLeod, 2016) calculated a power of 43.21% to 71.81% to detect an effect of .40 for pupil diameter.

#### Apparatus

The study took place in a sound-attenuated room with lights on. Participants were seated in front of a 24-inch screen (1,920 × 1,080 pixels, ca. 43.0 × 24.2 degrees of visual angle, 60-Hz refresh rate) at a distance of 70 cm, and their heads were stabilized by a chin rest. Binocular eye data were recorded using an SMI RED250 mobile system (SensoMotoric Instruments, Germany) with a temporal resolution of 250 Hz. The stimulus presentation program was written in PsychoPy (Peirce, 2007) using the Software Development Kit by SMI. There was a 9-point calibration procedure at the beginning of the practice and main block and a drift check before each task.

#### Procedure and task

Participants performed the alternate uses task, a divergent thinking task that is largely independent of sensory processing and therefore represents internally directed cognition (Benedek & Fink, 2019). This task is well suited to engage people for extended time periods in internally oriented cognition (Benedek, Christensen, Fink, & Beaty, 2019; Benedek, Mühlmann, Jauk, & Neubauer, 2013), which is necessary to ensure reasonable variation in the susceptibility to external distraction over time (Annerer-Walcher et al., 2018).

**Fig. 1:**
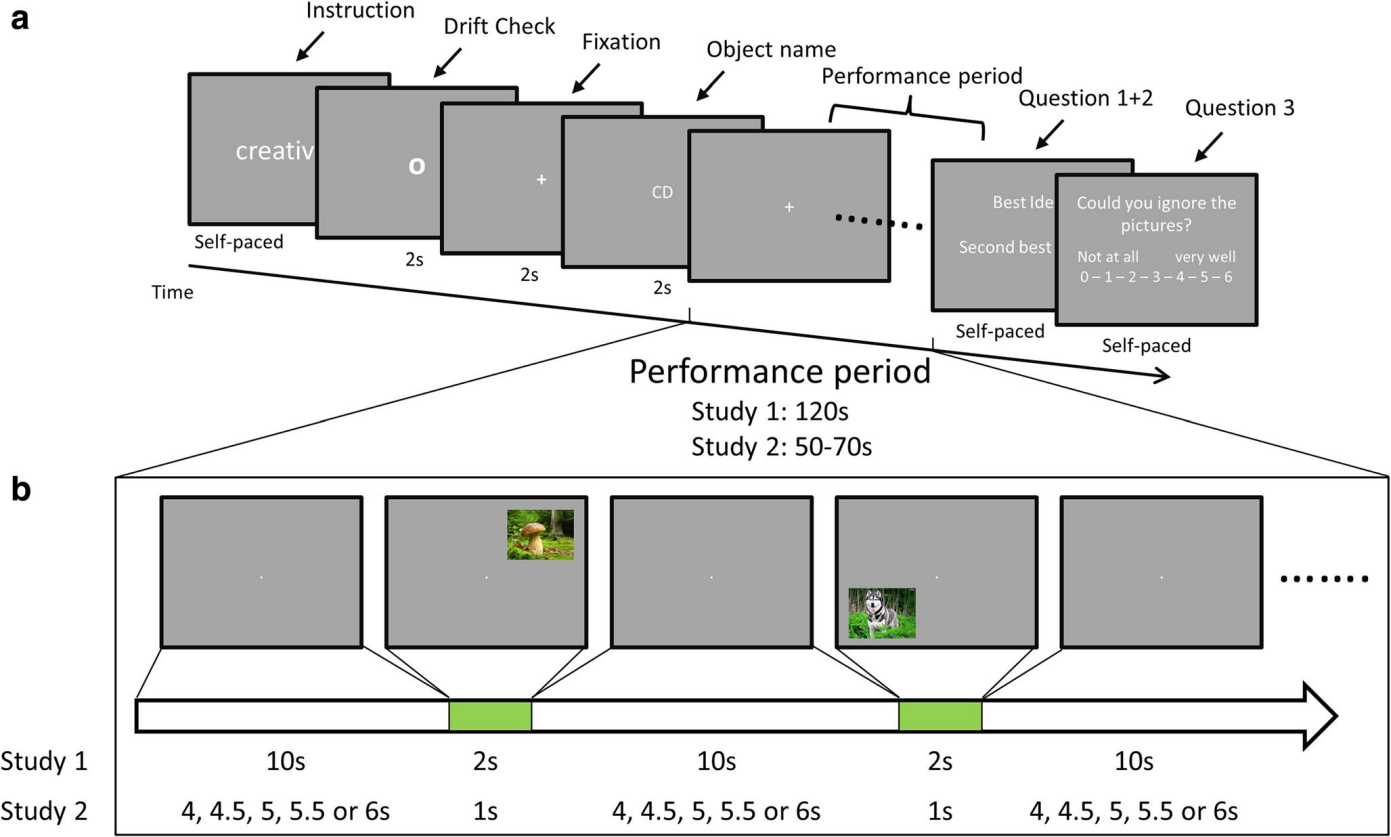
**a** Task procedure, and (**b**) time course of distractor presentation during the performance period of Study 1 and 2

Figure 1 illustrates the task procedure. At the beginning, participants received an object cue. They had been instructed to imagine all the creative uses for common household objects they could think of (see Fig. 1a). After a drift check and a 2-s fixation period (fixation cross of 30 pixels, ca. 0.67 degrees of visual angle in height in center of screen), the name of one of six common household objects (aluminum foil, paper clip, rubber band, toilet paper, paper cup, plastic bottle; practice task: credit card) appeared on the screen for 2 seconds. Then, participants had 120 s to find creative object uses without verbalizing them. They were told to find at least two creative object uses and to keep on trying to find more creative responses in the given time. Idea creativity tends to increase with time, but the task also becomes increasingly executively demanding as it requires to inhibit proactive interference by previous ideas (Beaty & Silvia, 2012). During this period, a fixation cross was presented in the center of the screen to ensure a constant reference of gaze position, while 10 irrelevant pictures (i.e., distractors) appeared every 12 s for 2 s each at one of the corners of the screen (see Fig. 1b and Stimulus Presentation section). We informed participants that the pictures were irrelevant and that they should maintain gaze on the middle of the screen throughout the experiment. This instruction was important to avoid gaze wandering outside of the screen during the extended task period, which would result in invalid eye-tracking data. Moreover, it generally represents a setting conducive to internal attention focus and creative thinking (Salvi & Bowden, 2016). Attention capture by distractor images during IDC has been successfully used as index of attentional focus in previous research (Buetti & Lleras, 2016). At the end of each task, participants were prompted to type in their best and second-best idea. Finally, participants had to indicate how well they were able to ignore the pictures during the task on an 8-point scale ranging from 0 (*not at all*) *to* 7 (*very well*). For better understanding of the following results, we recoded this variable to reflect self-reported distractor interference ranging from 0 (*no interference*) to 7 (*strong interference*).

Participants performed one practice task followed by six experimental tasks. In two additional baseline tasks, which were randomly intermixed with the experimental tasks, participants just had to remember the object’s name and to type it in at the end of the task. The baseline tasks thus required no idea generation, but involved the same presentation of distractor images. These baseline tasks served to assess task-independent effects of visual distraction on eye behavior.

After participants had completed all tasks, they answered questions regarding their perceived performance, distractor interference (i.e., difficulty to ignore distractors), and affective state during the tasks (see Table S1.1 in Supplemental Material 1). The study took about 45 min. It was part of a larger test session that included other paradigms and questionnaires that were not related to the present study. The total test session took 2.5 hours.

#### Stimulus presentation

The screen background was gray (RGB: 128, 128, 128), and all text and fixation stimuli were white. As distractors, we selected 80 color pictures from the International Affective Picture Scale (Lang, Bradley, & Cuthbert, 2008); picture numbers are listed in Supplemental Material 1). We selected pictures that were neutral in valence (*M* = 5.22, *SD* = 0.42, range: 4.21–6.09) and low in arousal (*M* = 3.53, SD = 0.40, range: 2.77–4.23), and 50% contained a human face. We scaled all pictures to 469 × 345 pixels (ca. 10.5 × 7.7 degrees of visual angle). To reduce effects of brightness and contrast, we normalized perceived brightness (mean (0.299*R + 0.587*G + 0.114*B) = 0.5; (Ridpath & Chisholm, 2000) and RMS contrast (root mean square contrast of 0.5) of all pictures.

For each task, 10 distractor pictures were selected quasirandomly, and each picture could appear only once in the entire experiment. The first distractor appeared 10 s after offset of the object cue. Then, every 12 seconds a picture appeared for 2 seconds at one of four positions: top left, top right, bottom right, and bottom left. The center of the picture was 345 pixels (ca. 7.7 degrees of visual angle) left or right, and 270 pixels (ca. 6.0 degrees of visual angle) above or below the center of the screen. A picture never appeared at the position of the preceding one. A task ended with the 2-second presentation of the last picture. During the whole task, a fixation cross was present in the center of the screen (height: 30 pixels, ca. 0.67 degrees of visual angle).

#### Data preprocessing

From raw pupil and gaze position data, we excluded samples recorded during a blink plus 2 (= 8 ms) additional samples at the beginning and the end of a blink to remove distorted data due to lid closure (percentage data discarded: *M* = 10.79, *SD* = 6.49, max = 27.06). We also excluded samples in which pupil diameter (PD) or fixation disparity (FD) were beyond their natural range (PD > 15 mm or PD < 1.8 mm, |FD| > mean pupil distance = 60 mm) or three standard deviations beyond an individual’s mean (percentage data discarded: *M* = 1.99, *SD* = 1.56, max = 7.09) to avoid data distortion due to measurement errors.

Saccades and microsaccades were determined using the Microsaccade Toolbox for R (Engbert, Sinn, Mergenthaler, & Trukenbrod, 2015), with λ = 6 for velocity threshold and a minimum duration of 8 ms (two samples). We considered only binocular saccades with a minimum overlap of one sample. We classified saccades smaller than or equal to 1.5° as microsaccades (Martinez-Conde, Macknik, Troncoso, & Hubel, 2009).

Pupil diameter and fixation disparity data were analyzed for time periods that were not a blink, saccade, or microsaccade. For each task, pupil diameter and fixation disparity were *z* transformed based on the 2-s fixation period at the beginning of that task.

We divided each task into 10 trials lasting from 10 s before distractor onset until distractor offset (12 s in total), and in each trial we selected the 2-s time segment preceding distractor onset for further analysis of eye behavior conditional to distractor capture. We counted onsets of saccades, microsaccades, and blinks within this 2-s time segment, and calculated medians of pupil diameter and fixation disparity for each segment. Saccades and microsaccades were still too rare (many segments with zero), so we collapsed saccades and microsaccades into a general saccade variable (Otero-Millan, Troncoso, Macknik, Serrano-Pedraza, & Martinez-Conde, 2008). We determined visual distraction by assessing whether participants looked at a picture (i.e., gaze registered in a region of interest defined by the picture size) or not at any time during the 2-s period of its presentation.

Task performance was scored with respect to the rated creativity of the generated ideas. Four experienced raters rated the creativity of all ideas (α = .68) on a 4-point scale ranging from 0 (*not creative*) to 3 (*very creative*; Diedrich, Benedek, Jauk, & Neubauer, 2015). Ratings were averaged across raters to yield a creativity score for each task.

#### Data analysis

We used generalized linear mixed-effects models because this method can be applied to a trial-level data set without the information loss that results from aggregating observations. This approach models both fixed and random effects, which offers more power and can handle unbalanced designs and missing data (Brysbaert & Stevens, 2018). We calculated generalized linear mixed-effects models using the *lme4* package (Bates, Machler, Bolker, & Walker, 2015) for R (R Core Team, 2016) and compared models using the *car* package (Fox & Weisberg, 2011). To assess significance of fixed effects, we applied the Satterthwaite approximation from the *lmerTest* package (Kuznetsova, Brockhoff, & Christensen, 2017).

To assess if eye behavior in a 2-s period prior to distractor onset predicts visual distraction (no/yes), we entered pupil diameter, fixation disparity, blink rate, and saccade rate as fixed effects. We added time of picture within trial (picture position) and time of trial within the experiment (trial position) as additional fixed effects to control for time-on-task effects. Regarding the random structure of our model, we included random intercepts for participants and pictures. We did not include random slopes because the model failed to converge.

### Results and discussion

#### Task performance

Participants were able to generate two ideas in all tasks, except for one participant who was not able to produce a second idea in one task. Mean rated creativity of the two idea responses was 1.14 (*SD* = 0.32, range: 0.06–1.75). This finding is consistent with previous research indicating that most divergent thinking responses are rated as not very creative (e.g., Diedrich et al., 2015). 34.52% (*SD* = 30.44) of distractor pictures captured attention, which suggests a substantial amount and variation in attentional focus despite successful idea generation.

#### Eye behavior

**Fig. 2:**
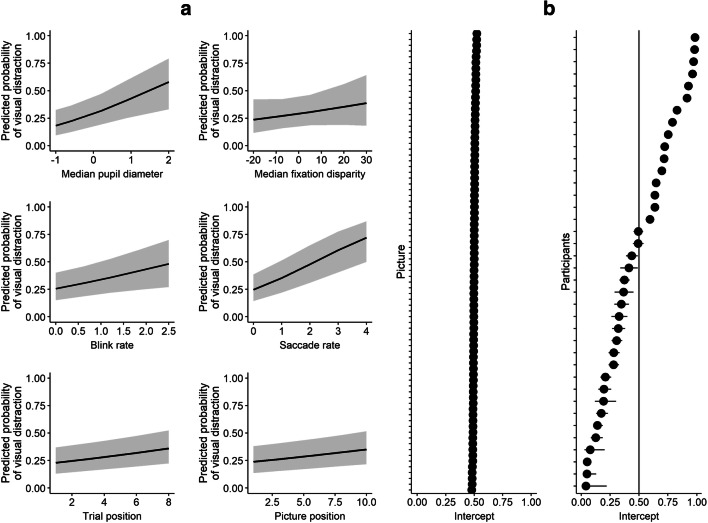
Illustration of the generalized linear mixed-effects model predicting visual distraction in Study 1. Fixed effects (**a**), random intercepts (**b**). Pupil diameter is centered to zero

Table 1 presents descriptive statistics of eye parameters separately for visual distraction (no/yes), and Table S2 in Supplemental Material 2 shows bivariate correlations between variables of the model. The generalized linear mixed-effects model analysis revealed significant fixed effects for pupil diameter, blink rate, saccade rate, trial position, and picture position (see Table 2 and Fig. 2). Fixation disparity did not contribute significantly to the prediction of visual distraction. Visual distraction was predicted by higher blink rate and saccade rate and stronger pupil dilation right before distractor onset. These results suggest that pupil, blinks, and saccade activity indicate transient variations in the susceptibility to visual distraction during IDC. There were also time effects. The probability to look at distractors increased from the first to the last task, and from the first trial (i.e., picture) to the last trial within tasks. This might reflect general exhaustion effects consistent with the notion that divergent thinking tasks become increasingly executively demanding (Beaty & Silvia, 2012), which results in a decline of the capacity to maintain sustained attention on the internal task and to ignore distractors.

**Table 1.** Descriptive statistics of average eye behavior 2 s before distractor onset depending on visual distraction (no/yes)

	Study 1 Visual distraction				Study 2 Visual distraction			
	No		Yes		No		Yes	
Variable	*M*	*SD*	*M*	*SD*	*M*	*SD*	*M*	*SD*
Pupil diameter	4.19	0.17	4.25	0.24	4.50	0.10	4.52	0.10
Fixation disparity	0.30	2.46	−0.01	3.00	1.59	2.25	1.05	2.25
Blink rate	0.40	0.13	0.46	0.13	0.34	0.11	0.40	0.11
Saccade rate	0.36	0.17	0.55	0.20	0.37	0.18	0.49	0.18

*Note*. Distraction rate was 34.52% in study 1 and 39.37% in Study 2

**Table 2.** Generalized linear mixed-effects models predicting visual distraction in Study 1 and Study 2

	*b*	*SE*	*z*	*p*	95% CI
**Study 1** (*N* = 38, observations = 2,219)					
(Intercept)	**−2.05**	0.38	−5.32	<.001	−2.80, −1.29
Pupil diameter	**0.60**	0.20	3.01	.003	0.21, 1.00
Fixation disparity	0.01	0.01	1.02	.309	−0.01, 0.04
Blink rate	**0.40**	0.16	2.46	.014	0.08, 0.71
Saccade rate	**0.52**	0.10	5.21	<.001	0.32, 0.71
Trial position	**0.09**	0.03	3.46	.001	0.04, 0.14
Picture position	**0.06**	0.02	2.93	.003	0.02, 0.10
*R*^2^m = 5%					
*R*^2^c = 57%					
χ^2^ = 61.05, *df* = 6, *p* < .001					
**Study 2** (*N* = 144, observations = 10,209)					
(Intercept)	**−1.48**	0.19	−7.90	<.001	−1.85, −1.11
Pupil diameter	**0.38**	0.09	4.31	<.001	0.21, 0.55
Fixation disparity	−0.01	0.01	−2.78	.005*	−0.02, −0.01
Blink rate	**0.45**	0.07	5.98	<.001	0.30, 0.59
Saccade rate	**0.38**	0.04	8.93	<.001	0.30, 0.47
Trial position	**0.05**	0.01	3.95	<.001	0.02, 0.07
Picture position	**0.06**	0.02	3.22	.001	0.02, 0.10
*R*^2^m = 3%					
*R*^2^c = 48%					
χ^2^ = 162.17, *df* = 6, *p* < .001					

*Note.* 95% CI = 95% confidence intervals. *R*^2^m denotes variance explained by the fixed effects and *R*^2^c denotes variance explained by fixed and random effects

*Fixation disparity was significant in the model, however removing fixation disparity did not harm model fit significantly

In the baseline tasks, visual distraction was predicted by stronger pupil dilation and smaller fixation disparity. Blink rate and saccade rate did not contribute to the model in baseline tasks (see Supplemental Material 1), suggesting that they are specific to predict visual distraction during goal-directed IDC.

Regarding random effects, random intercepts for pictures were not significant (variance = 0.02, *SD* = 0.15, χ^2^ = 0.24, *p* = .621), suggesting that the specific characteristics of distractor pictures (e.g., color, content) had no significant influence on whether they caught attention or not. Specifically, the presence of faces had no effect on the distraction rates as 31.2% of the pictures without and 32.5% of the pictures with faces were looked at (χ^2^ = 0.548, *p* = .459). There was a large effect of random intercepts for participants (variance = 3.97, *SD* = 1.99, χ^2^ = 798.13, *p* < .001; see Fig. 2). Participants varied strongly in their distraction rate, suggesting considerable individual difference in the capacity to maintain sustained internally directed attention despite distraction (see Supplemental Material 1).

To further explore the effect of visual distraction on task performance, we examined relationships between distraction rate, self-reported distractor interference, and response creativity. Relations were analyzed at the within-subject level (across tasks) and at the between-subject level (across participants). Self-reported distractor interference tended to be higher for tasks with higher distraction rates (mean correlation across participants: *M* = .34, *SD* = .40, range: −.81 to .92), but there was no significant relationship at the between-subjects level (*r* = .18, *p* = .285). Interestingly, distraction rate and self-reported distractor interference was not related to response creativity, neither at the within-subject level (*M* = .07, *SD* = .39, range: −.48 to .84; *M* = -.06, *SD* = .42, range: −.82 to .72), nor at the between-subject level (*r* = −.04, *p* = .814; *r* = −.11, *p* = .521).

In sum, the extended IDC task allowed considerable rates of visual distraction (36%), and higher susceptibility to visual distraction was predicted by higher pupil diameter, blink rate, and saccade rate. Distraction rates were only weakly related to self-reported distraction interference and were not systematically associated with task performance.

As a limitation of this study, distractor pictures appeared at a fixed interval of 12 s, which makes them predictable and thus less distracting. From research on mind wandering, we know that people can adapt their mind wandering to task demands (Seli et al., 2018). Moreover, Unsworth, Robison, and Miller (2018) found that pupil diameter increases when expecting a relevant stimulus, a result to which the authors refer as *increase in intrinsic alertness*. In their study, this preparatory increase of pupil diameter was somewhat smaller for longer ISIs (8 s compared with 2 s). Even in trials with the slowest responses, pupil diameter increased in anticipation of the next stimulus (although not as much as in trials with the fastest responses; Unsworth et al., 2018). As distractor onsets were fixed in Study 1, differences in eye parameters could be the result of successful prediction of distractor onsets. Therefore, in Study 2, we tested whether our findings are robust against less predictable picture onsets. Specifically, we varied the time between distractors (4–6 s) and the number of distractors per task (7–11), as well as total task duration (50–70 s). Furthermore, we increased sample size in Study 2 to increase statistical power of within- and between-subject effect analyses.

## Study 2

### Method

#### Participants

The final sample consisted of 144 adults (105 female), ages 19 to 48 years (*M* = 24.33, *SD* = 4.76). They participated for payment (€10/h). Most participants were students (88.2%). One hundred and twenty-eight participants had normal vision; 16 participants had corrected-to-normal vision (soft contact lenses) and reported no strabismus or other medical conditions affecting vision. All participants gave written informed consent. We excluded thirteen additional participants from analyses (nine female; mean age = 24.38 years). Five had excessive missing data (>50%) due to eye-tracker malfunction. Eight participants were able to ignore all pictures, leaving no variance in susceptibility to visual distraction to analyze. With the final sample of 144 participants and 72 pictures per participant, a total of 10,209 usable observations were collected. Post hoc power simulations using the *powerSim* function of the *simr* package (Green & MacLeod, 2016) calculated a power of 92.89% to 100% to detect an effect of .40 for pupil diameter.

#### Procedure, task, and apparatus

The same eye tracker was used as in Study 1. The paradigm of Study 2 was largely identical to Study 1 (see Fig. 1), except for a few small changes mainly targeted to increase the unpredictability of distractor onsets and to increase the power of analyses: (1) Participants performed eight experimental tasks instead of six. (2) We reduced task durations from 120 s to a varying duration of 50 to 70 s. (3) Within tasks, eight to 12 distractor pictures were presented instead of 10. (4) They were presented for 1 s instead of 2 s, (5) with a reduced and varying interstimulus interval between distractors (4, 4.5, 5, 5.5, 6 s) instead of a fixed interval of 10 s. Pictures were quasirandomly selected from the 80 pictures of Study 1, and interstimulus-intervals were randomized. We excluded the last picture of each task from analysis, because data acquisition ended during picture presentation, cutting short the time in which looking at a picture would have been possible. In total, 72 picture presentations per participant were considered in the analyses. The reduced task duration further limits the risk of task-unrelated thoughts, as 1 minute has been estimated as the minimum time needed to ensure that most participants produce at least two original ideas (Benedek et al., 2013).

Study 2 was part of a larger test session. The first part was a 45-minute online session (at home using LimeSurvey (LimeSurvey Project Team/Carsten Schmitz, 2015) including questionnaires on personality traits, mind wandering, and psychological health. The second part was a 3.5-hour session in our lab, with breaks. In the lab session, participants performed cognitive tests (see Supplemental Material 2), the present paradigm and two further eye-tracking experiments, not related to the present manuscript.

#### Data preprocessing and analysis

Data preprocessing and analysis was identical to Study 1. On average, 12.40% of samples were excluded due to blinks (*SD* = 9.14, max = 42.06), and 3.18% due to pupil diameter and fixation disparity outliers (*SD* = 2.38, max = 10.71). The number of valid segments per participant ranged from 68 to 72. The creativity of generated ideas was rated by eight experienced raters to ensure good interrater reliability (α = .74).

### Results and discussion

#### Task performance

Participants were able to name a best idea in all tasks and a second-best idea in 96% of tasks. Mean originality of the two ideas was rated 1.03 (*SD* = 0.21, range: 0.49–1.82). On average, participants looked at 28.31 out of 72 distractor pictures (*SD* = 20.52, range: 1–71), which corresponded to an average distraction rate of 39.37% (only segments with available eye data were considered), similar to Study 1.

#### Eye behavior

**Fig. 3:**
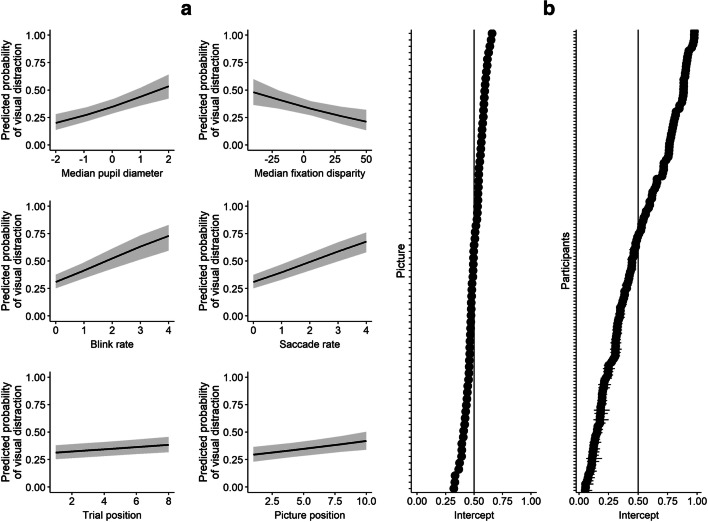
Illustration of the generalized linear mixed-effects model predicting visual distraction in Study 2. Fixed effects (**a**), random intercepts (**b**). Pupil diameter is centered to zero

Table 1 presents descriptive statistics of eye parameters separately for visual distraction (no/yes), and Table S2 in Supplemental Material 2 shows bivariate correlations between variables of the model across samples. We used the same model in the generalized linear mixed-effects analysis as in Study 1. The final model revealed significant fixed effects for pupil diameter, blink rate, saccade rate, trial position, and picture position (see Table 2 and Fig. 3), while fixation disparity did not contribute to the model. As in Study 1, the probability of visual distraction increased with higher pupil diameter, saccade rate, and blink rate. A graphical analysis of the time course relative to distractor onset is given in the Supplemental Material 3. It illustrates that the observed distractibility effects are robust across the 2-s time window preceding distractor onset.

Moreover, similar to Study 1, visual distraction further increased with time within and across tasks. Thus, the pattern of fixed effects was identical to Study 1 (see Table 2). This underlines the robustness of eye parameters as predictors of visual distractibility despite reduced predictability of picture onsets.

Random intercepts for pictures were significant (variance = 0.14, *SD* = 0.38, χ^2^ = 113.17, *p* < .001), suggesting that characteristics of distractor pictures (e.g., color, content) had some influence on whether they were looked at or not (see Fig. 3b). There was again a large effect of random intercepts for participants (variance = 2.68, *SD* = 1.64, χ^2^ = 2901.68, *p* < .001; see Fig. 3b). Yet the presence of faces again had no effect on distraction rates: 38.4% of the pictures without and 39.2% of the pictures with faces were looked at (χ^2^ = 0.626, *p* = .429).

Participants varied strongly in their distraction rate. We tested the idea that individual differences in susceptibility to visual distraction may be related to cognitive abilities. Exploratory analysis showed that attention capacity, executive functioning, creative potential, and openness measures only marginally predicted the distraction rate in the divergent thinking task (see Supplemental Material).

**Fig. 4:**
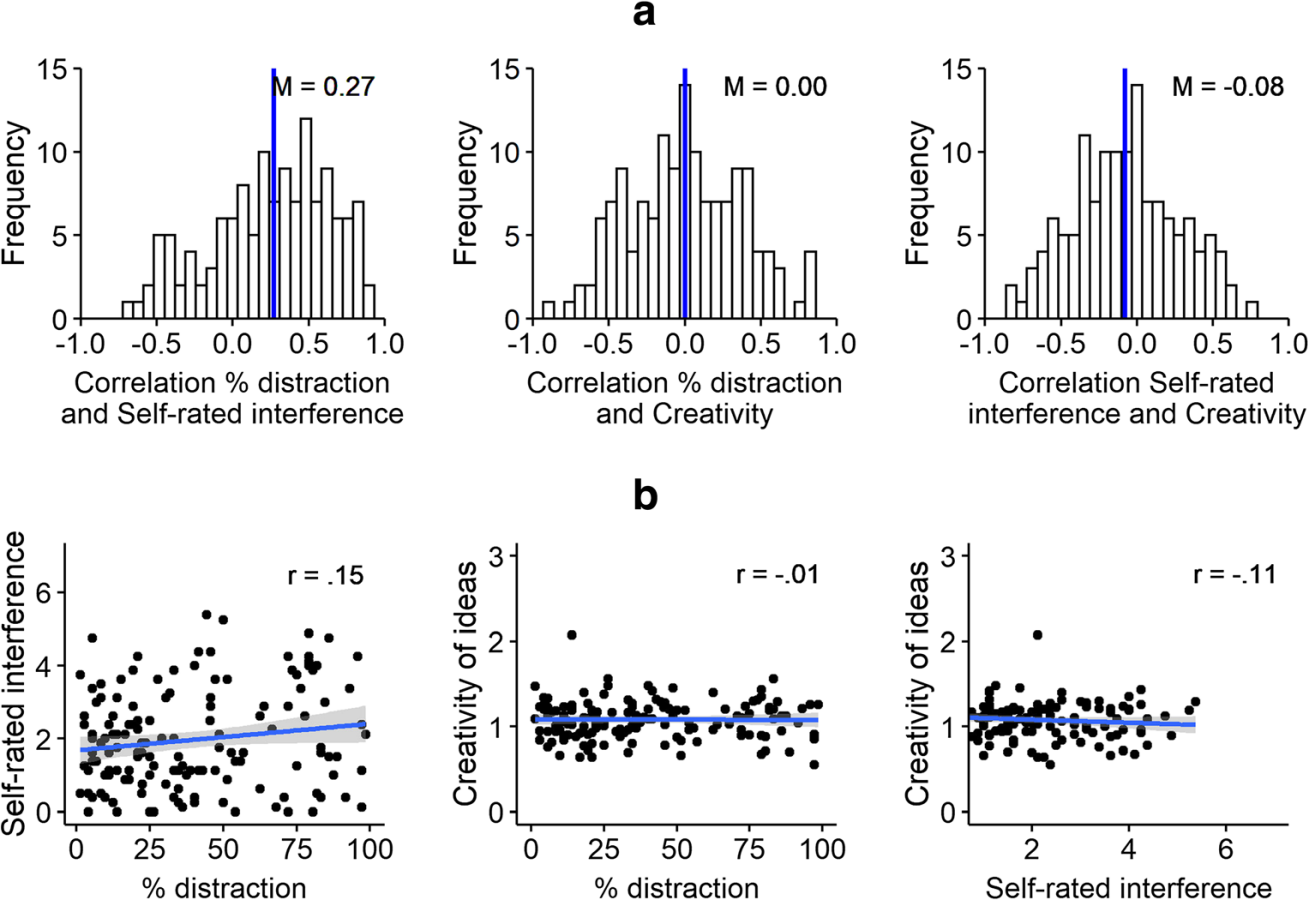
Within-subject (**a**) and between-subject (**b**) analyses. Correlations between distraction rates (% distraction), self-reported distractor interference (self-rated interference), and task performance (rated creativity of responses) in Study 2. Further individual differences analyses are presented in the Supplemental Material 2

We found the same (lack of) correlations between distraction rate, self-reported distractor interference, and task performance (creativity of ideas) at the within-subject and between-subject level as in Study 1. Self-reported distractor interference tended to be higher in tasks with higher distraction rate (*M* = .27, *SD* = .40, range: −.66 to .97) and for participants with higher distraction rate (*r* = .15, *p* = .064). Yet distraction rates and self-reported distractor interference did not predict task performance, either at the within-subject level (*M* = .00, *SD* = .37, range: −.90 to .86 and *M* = .08, *SD* = .35, range: −.79 to .85; see Fig. 4a) or the between-subject level (*r* = −.01, *p* = .936 and *r* = −.11, *p* = .198; see Fig. 4b).

These results provide further support that susceptibility to visual distraction during idea generation had no overall effects on idea generation performance. It is possible that some participants’ performance suffered from visual distraction, whereas others’ performance was unaffected or may have even benefitted. For example, investigations of the relationship between mind wandering and creativity have revealed mixed findings: Mind wandering (spontaneous task-unrelated internal attention focus) during idea generation was found to be detrimental for task performance (Hao, Wu, Runco, & Pina, 2015), but had no effect when it occurred during incubation periods (Smeekens & Kane, 2016); yet trait mind-wandering propensity was shown to be positively related to creative task performance (Agnoli, Vanucci, Pelagatti, & Corazza, 2018). To investigate potentially different effects of spontaneous external and internal attention focus (mind wandering) on idea generation performance more closely, future studies should assess both simultaneously.

## General discussion

This study investigated eye behavior as indicator of within-task susceptibility to visual distraction during internally directed cognition. Extended task performance of creative idea generation implied substantial rates of visual distraction (35% and 39%, for Study 1 and 2, respectively). This enabled a powerful analysis of eye behavior as predictor for the oculomotor capture by distractors. Visual distraction was preceded by more blinks, more saccades, as well as higher pupil dilation. Results replicated across two studies with different predictability of distractor onsets, underlining the robustness of the findings.

The present findings are strikingly similar to those observed during mind-wandering episodes. In mind wandering studies (internal) distraction during an external task is also preceded by larger pupil diameter, more blinks, and more saccades (Franklin, Mrazek, Broadway, & Schooler, 2013b; Konishi et al., 2017; Reichle et al., 2010; Smallwood et al., 2011; Smilek et al., 2010; Unsworth & Robison, 2018). Although attentional focus of distraction and task are reversed in this case, both mind wandering studies and the present paradigm share failures in maintaining task focus in face of distraction. The consistent eye behavior pattern observed during mind wandering and visual distraction may hence reflect incidents of failed or reduced task focus during extended task engagement.

While reduced task focus typically leads to impaired task performance in mind-wandering studies, no impairments were observed in the present study. In part, this may be because mind-wandering studies assess performance more sensitively (i.e., response time per trial vs. response quality per task; Smallwood et al., 2011). Moreover, in the context of creative performance, distraction and reduced control are discussed to have negative as well as positive effects (Baird et al., 2012). This may explain why we observed no correlation between distraction rates and creative task performance.

As in mind-wandering episodes, visual distraction during an internal task was preceded by larger pupil diameter and more blinks. Increases in pupil diameter are known to reflect increases in cognitive load (Kahneman & Beatty, 1966; Piquado, Isaacowitz, & Wingfield, 2010). Maintaining sustained internally directed attention in face of distraction may become increasingly cognitively taxing, as evidenced by increases in distraction rates over time. In moments of excessive cognitive demands (as indicated by large pupil diameter and potentially high compensatory blink rate), people may sometimes fail to maintain the internal focus of attention and get distracted by salient sensory stimulation, thereby interrupting the internal stream of thought. Given, the similar oculometric correlate to visual distraction and mind wandering, future studies should explore whether visual distraction and mind wandering during an internal task can actually be discriminated at the level of eye behavior.

The heightened saccadic activity right before visual distraction could represent reduced coupling to task-relevant imagination processes and increased spontaneous sampling of visual information (even when no relevant information is available). Searching for visual information in the external environment is associated with more saccades than focusing on something (Ehrlichman & Micic, 2012). In a state of increased spontaneous sampling of visual information, it can be assumed that people are more likely to notice and attend to visual distractors. Yet the current results oppose the notion that susceptibility to visual distraction is simply akin to a more external focus. Several studies have reported more or longer blinks and larger pupil diameter during IDC compared with EDC (Annerer-Walcher et al., 2018; Benedek, Stoiser, et al., 2017b; Salvi et al., 2015; Savage, Potter, & Tatler, 2013; Smilek et al., 2010; Walcher et al., 2017) and have argued that blink rate is reduced in states of heightened focus to sensory information (Shin et al., 2015; Shultz, Klin, & Jones, 2011), whereas higher blink rate may facilitate the shielding of an internal train of thought by direct attenuation of visual input (Benedek, Stoiser, et al., 2017b; Salvi et al., 2015; Walcher et al., 2017). Moreover, larger pupil diameter is commonly viewed as an indicator of increased mental load (Kahneman & Beatty, 1966; Piquado et al., 2010), and goal-directed IDC often involves high workload as all relevant information must be kept in working memory and cannot be externally retrieved (Benedek et al., 2011; Benedek, Schickel, et al., 2014b). Notably, studies comparing internal and external tasks differ fundamentally from the present design. In the former, goal-directed IDC is compared with goal-directed EDC and distraction during the internal task is typically minimal (Benedek, Stoiser, et al., 2017b; Walcher et al., 2017). Here, we studied how variation in the susceptibility to visual distraction *during* an internal task is reflected in eye behavior. And the present findings showed that eye behavior differences between low and high susceptibility to visual distraction during an internal task are not consistent with eye behavior differences between goal-directed IDC and goal-directed EDC. Therefore, taken together, the oculometric findings suggest that increased visual distractibility does not simply correspond to a more external attention state, but are rather in line with a reduced/failed task focus interpretation.

In the present study, distraction rates varied considerably across participants. Moreover, we also observed large individual differences in the relationship between distraction rate and creative task performance (range: −.90 to .86). While these values may just reflect expected variability around zero, they might also suggest that visual distraction could be both beneficial for some people and for others detrimental for the idea generation process. It is assumed that both spontaneous and controlled processes are relevant to creative cognition (Beaty, Silvia, Nusbaum, Jauk, & Benedek, 2014; for reviews, see Benedek & Jauk, 2018, 2019; Chrysikou, 2018). On the one hand, there is robust evidence that higher executive capacity predicts higher creative task performance (Benedek, Jauk, Sommer, Arendasy, & Neubauer, 2014a; Silvia, 2015), but on the other hand, creativity is sometimes found to benefit from states of reduced control (e.g., Benedek, Panzierer, Jauk, & Neubauer, 2017a; Gable, Hopper, & Schooler, 2019; Radel, Davranche, Fournier, & Dietrich, 2015). This may be related to the ambiguous role of task-irrelevant information for creative thought: Looking at distractor pictures could have triggered spontaneous autobiographical or semantic memories (Faber & D’Mello, 2018), which may sometimes have promoted performance by inspiring novel associations for possible uses and sometimes may have hampered performance by simply interrupting the ongoing internal train of thought.

We further observed that distraction rates increased with time. We presumed that this might be due to general fatigue/exhaustion effects. Processing of irrelevant information was shown to be increased when working memory load is high as well as for people of lower working memory capacity, which is consistent with the assumption of load theory that cognitive control is needed to keep clear processing priorities in the face of potential distraction (de Fockert, 2013). Interestingly, however, studies examining visual distraction during EDC tasks have previously reported reduced oculomotor capture by distractors with time suggesting habituation effects (Bonetti & Turatto, 2019; Godijn & Kramer, 2008). This may point to an interesting distinction of the role of visual distraction between IDC and EDC tasks. Maybe, having an external attention focus allows more effective strategies for deciding which perceptual information should be attended and which should be ignored. Moreover, studies with EDC tasks usually use comparatively short tasks compared with our extended IDC task. In the context of such short tasks, training effects may exceed fatigue effects and thereby result in increasingly lower working memory load and thus more effective distractor inhibition.

The present study showed that it is difficult to maintain sustained internal attention focus for 1–2 minutes, as visual distractors frequently catch our attention. Visual distraction was predicted by pupil dilation, and increased blink and saccade rate. These results favor a reduced/failed task focus interpretation over an external attention interpretation as underlying mechanism in states of increased susceptibility to visual distraction. Moreover, the findings highlight that eye behavior represents a sensitive indicator of attentional focus within complex mental tasks.

## Electronic supplementary material

ESM 1 (DOCX 20 kb)

ESM 2 (DOCX 45 kb)

ESM 3 (DOCX 376 kb)
