# Supplementary material for: Eye behavior predicts susceptibility to visual distraction during internally directed cognition
Source: Atten Percept Psychophys. 2020 Jun 4;82(7):3432–44. doi: 10.3758/s13414-020-02068-1 (PMC7536161; doi:10.3758/s13414-020-02068-1)
Supplement: Supplementary file 1 — (DOCX 20 kb) [file 13414_2020_2068_MOESM1_ESM.docx]

# Supplemental Material 1 of manuscript:

**Eye behavior predicts susceptibility to visual distraction during internally directed cognition**

Additional information on stimulus material and side analyses:

**List of IAPS distractor images:**

Image numbers:

Main tasks (*N* = 80): 1670, 2002, 2020, 2038, 2101, 2102, 2214, 2215, 2235, 2305, 2308, 2357, 2372, 2382, 2390, 2393, 2397, 2400, 2411, 2489, 2512, 2513, 2514, 2575, 2579, 2593, 2594, 2597, 2690, 2749, 2850, 2890, 4500, 4510, 4573, 5120, 5250, 5395, 5471, 5520, 5531, 5532, 5534, 5535, 7002, 7003, 7019, 7030, 7032, 7036, 7038, 7042, 7043, 7052, 7057, 7081, 7096, 7161, 7165, 7192, 7205, 7207, 7211, 7242, 7290, 7300, 7354, 7365, 7487, 7493, 7495, 7546, 7547, 7595, 7710, 8121, 8312, 8465, 9210, 9401.

Practice task (*N* = 10): 2221, 2383, 2506, 4274, 5510, 5530, 7050, 7179, 7233, 9260.

Valence: *M* = 5.22, range: 4.21 – 6.09.

Arousal: *M* = 3.53, range: 2.77 – 4.23.

| Table S1.1  *Descriptive statistics of questions asked after end of Study 1.* | | | |  |
| --- | --- | --- | --- | --- |
| Question | Scale | *M* | *SD* | |
| During idea generation, I was distracted by thoughts, daydreams and/or feelings. Internal information sources | 1 = never  6 = very often | 3.00 | 1.34 | |
| During idea generation, I was distracted by external stimuli (e.g. sounds, flicker, itches). External information sources | 1 = never  6 = very often | 2.37 | 1.50 | |
| It was important for me to show good performance. | 1 = agree  6 = disagree | 4.00 | 1.19 | |
| The idea generation task was fun. | 1 = agree  6 = disagree | 4.00 | 1.19 | |
| The idea generation task was demanding. | 1 = not at all  6 = very | 3.13 | 1.38 | |
| Right now, I feel … | 1 = very tired  6 = very awake | 3.24 | 1.34 | |
| The pictures were distracting. | 1 = agree  6 = disagree | 2.87 | 1.32 | |
| Ignoring the pictures was easy for me. | 1 = agree  6 = disagree | 4.00 | 1.32 | |
| How often did you look at pictures? | 1 = never  6 = always | 3.26 | 1.16 | |
| The pictures made idea generation task… | 1 = harder  6 = easier | 3.11 | 0.83 | |
| How often did the pictures help you find new ideas? | 1 = never  6 = always | 1.76 | 1.08 | |
| *Note*. Participants answered those questions after completing all tasks. Due to restricted time, this questionnaire was not presented in Study 2. *N* = 38. | | | |  |

| Table S1.2  *Bivariate correlations between variables of the model across samples.* | | | | | | | |
| --- | --- | --- | --- | --- | --- | --- | --- |
| Study 1 | | | | | | | |
|  |  | 1 | 2 | 3 | 4 | 5 | 6 |
| 1 | Visual distraction (0=no, 1=yes) |  |  |  |  |  |  |
| 2 | Pupil diameter | .05 |  |  |  |  |  |
| 3 | Fixation disparity | .01 | .06 |  |  |  |  |
| 4 | Blink rate | .04 | .11 | .08 |  |  |  |
| 5 | Saccade rate | .19 | -.03 | .00 | .14 |  |  |
| 6 | Trial position | .07 | -.07 | .02 | .04 | .04 |  |
| 7 | Picture position | .03 | -.16 | .00 | -.05 | -.02 | .00 |
|  | | | | | | | |
| Study 2 | | | | | | | |
|  |  | 1 | 2 | 3 | 4 | 5 | 6 |
| 1 | Visual distraction (0=no, 1=yes) |  |  |  |  |  |  |
| 2 | Pupil diameter | .05 |  |  |  |  |  |
| 3 | Fixation disparity | .07 | -.13 |  |  |  |  |
| 4 | Blink rate | .11 | .11 | .17 |  |  |  |
| 5 | Saccade rate | .21 | .05 | .01 | .02 |  |  |
| 6 | Trial position | .02 | -0.12 | .06 | .04 | .03 |  |
| 7 | Picture position | .04 | -0.10 | .01 | -.07 | .00 | .01 |
| *Note.* | | | | | | | |

| Table S1.3  *Study 1, baseline tasks, descriptive statistics of average eye behavior 2s before distractor onset depending on visual distraction (no/yes).* | | | | |
| --- | --- | --- | --- | --- |
|  | Visual distraction | | | |
|  | No | | Yes | |
| Variable | *M* | *SD* | *M* | *SD* |
| Pupil diameter | 3.91 | 0.25 | 4.04 | 0.25 |
| Fixation disparity | 0.22 | 3.87 | -0.52 | 2.72 |
| Blink rate | 0.31 | 0.16 | 0.42 | 0.18 |
| Saccade rate | 0.25 | 0.20 | 0.25 | 0.24 |
| *Note*. | | | | |

| Table S1.4  *Study 1, baseline tasks, bivariate correlations between variables of the model across all samples.* | | | | | | | |
| --- | --- | --- | --- | --- | --- | --- | --- |
|  | Variable | 1 | 2 | 3 | 4 | 5 | 6 |
| 1 | Visual distraction (0=no, 1=yes) |  |  |  |  |  |  |
| 2 | Pupil diameter | .09 |  |  |  |  |  |
| 3 | Fixation disparity | .08 | .09 |  |  |  |  |
| 4 | Blink rate | .13 | .12 | .07 |  |  |  |
| 5 | Saccade rate | .18 | .08 | .02 | .25 |  |  |
| 6 | Trial position | -.04 | -.10 | .11 | -.09 | .02 |  |
| 7 | Picture position | .07 | -.20 | -.04 | .08 | .09 | -.01 |
| *Note.* | | | | | | | |

| Table S1.5  *General linear mixed models predicting distractor capture in baseline tasks of Study 1.* | | | | | |
| --- | --- | --- | --- | --- | --- |
|  | *b* | *SE* | *z* | *p* | 95% CI |
| Baseline tasks  (*N* = 38, samples = 742) |  |  |  |  |  |
| (Intercept) | **-2.41** | 0.65 | -3.71 | <.001 | -3.48, -1.13 |
| Pupil diameter | **1.11** | 0.38 | 2.96 | .003 | 0.38, 1.85 |
| Fixation disparity | -0.04 | 0.03 | -1.40 | .163 | -0.08, 0.01 |
| Blink rate | 0.57 | 0.35 | 1.65 | .099 | -0.11, 1.25 |
| Saccade rate | 0.00 | 0.26 | 0.01 | .992 | -0.48, 0.49 |
| Trial position | **-0.20** | 0.07 | -2.72 | .006 | -0.35, -0.06 |
| Picture position | **0.15** | 0.05 | 3.21 | .001 | 0.06, 0.24 |
| R^2^m = 8% |  |  |  |  |  |
| R^2^c = 71% |  |  |  |  |  |
| χ2 =34.02, df = 6, p < .001 | | | | |  |
| *Note*. 95% CI = 95% confidence intervals. R^2^m denotes variance explained by the fixed effects and R^2^c denotes variance explained by fixed and random effects. | | | | | |
