# Supplementary material for: Eye behavior predicts susceptibility to visual distraction during internally directed cognition
Source: Atten Percept Psychophys. 2020 Jun 4;82(7):3432–44. doi: 10.3758/s13414-020-02068-1 (PMC7536161; doi:10.3758/s13414-020-02068-1)
Supplement: Supplementary file 2 — (DOCX 45 kb) [file 13414_2020_2068_MOESM2_ESM.docx]

# Supplemental material 2 of manuscript:

# Eye behavior predicts susceptibility to visual distraction during internally directed cognition

# Exploratory analysis of individual differences in the distraction rate during idea generation

In Study 2, we found huge individual differences in the number of attended pictures.

There are two major approaches to the study of human intellectual performance. The first focuses on the effect of personality and individual differences, and the second attempts to develop general laws of cognitive psychology or information processing. Although these two approaches rarely are combined, it is difficult to find an example of cognitive performance that is not better understood by a combination of both areas. (Humphreys & Revelle, 1984, p. 153)

Following this suggestion, we explored individual differences in the number of attended pictures found in Study 2 using a broad battery of measures assessed for other studies within the same test session.

## Method

### Measures for exploratory analysis

As a dependent variable, we calculated the proportion of pictures a participant looked at relative to all pictures with eye tracking data for this participant.

We used a broad range of measures assessing possible predictors of susceptibility to distraction. All questionnaires were completed online through LimeSurvey prior to the lab session. In the lab session, operation span task, arrow flanker task, and antisaccade task were administered using PsychoPy. Letter sets, number series, and paper folding were administered in the lab and automatically timed using LimeSurvey. Verbal fluency task was performed via paper and pencil.

#### Working memory capacity.

*Operation span task*. We measured working memory capacity with an operation span task (von der Malsburg, 2015). Participants memorized sequences of 3 to 7 letters, which were randomly selected without replacement from a set of 21 consonants. After each letter, a compound arithmetic equation appears which participants need to verify [e.g. ( 8 - 2 ) × 4 = 28]. Half of the equations were true. At recall, participants typed in the letters in correct order. We used the partial credit unit as score (max. 1) which is the mean proportion of letters within an item that were recalled correctly.

#### Attention.

*Arrow Flanker task*. To assess the ability to constraint their attention to targets amidst visual targets, we used an arrow flanker task (Eichele et al., 2008; Jangraw, 2015). Five arrows (“<” and “>”) were presented on a vertical line. Participants indicated the direction of the central arrow by pressing the corresponding arrow key. On congruent trials, all 5 arrows pointed in the same direction. On incongruent trials, the distractors pointed in the opposite direction as the target. We used the performance difference between congruent and incongruent as measure (higher values equal less interference by distractors).

*Antisaccade task*. To assess the ability to override prepotent responses with another response, we used the Antisaccade task (based on Kane, Bleckley, Conway, & Engle, 2001). Participants identified a letter (B, R, or P) that appeared left or right of the screen center for 100ms. A flashing cue (“=”), at the same position (prosaccade) or the opposite position of the target (antisaccade) appeared right before the target. Participants reported the letter via a number keyboard (1 = B, 2 = R, 3 = P). We used the difference in error rate between antisaccade and prosaccade as measure (higher values equal more errors during antisaccade condition).

*SIPI poor attentional control subscale.* To assess attention problems in everyday life, we used the poor attentional control subscale (α = .82) of the Short imaginal processes inventory (SIPI, Huba, Aneshensel, & Singer, 1981). Participants answered 15 items on a 5-point scale from 1 = *not at all/completely untypical for me* to 5 = *completely/very typical for me*. An example item: “Faced with a tedious job, I notice all the other things that I could be doing.” Scores were summed.

*ASRS*. We screened ADHD symptoms with the screening test (α = .35) of the Adult ADHD Self-Resport Scale v1.1 (ASRS, World Health Organization, 2004). The six screening items screened the whole spectrum of ADHD symptoms (e.g. mindlessness, restlessness) on 5-point scales ranging from 1 = *never* to 5 = *always*. Scores were summed.

***Executive functions.***

*Letter sets*. We assessed verbal intelligence with the first set (15 items) of the letter sets task (Ekstrom, French, Harman, & Derman, 1976). In each item, five four-letter strings were presented. One had to find the common rule and mark the string that did not match this rule. We used the number of solved items within 4 minutes as a measure (max = 15, α = .68).

*Number series*. We assessed numerical intelligence with the number series task (Amthauer, Brocke, Liepmann, & Beauducel, 2001). Participants typed in the next number in a sequence of seven numbers, which were connected through a hidden rule. We used the number solved items within 4.5 minutes as a measure (max = 15, α = .88).

*Paper folding*. We assessed figural intelligence with the first item set of the paper folding task (Ekstrom et al., 1976). Per item, folding of a square paper is depicted stepwise (2 to 4 folds per item). A small circle on the final folded paper indicates where it is pierced through by a pen. Participants had to determine how the paper looks when unfolded again and select the correct response from a set of 5 options. We used the number of solved items within 3 minutes as a measure (max = 10, α = .71).

*Verbal fluency*. We assessed verbal retrieval ability with the verbal fluency test (Silvia, Beaty, & Nusbaum, 2013). Participants had two minutes per item. In the first two items, they named as many words starting with “F” and “S” as possible. In item 3 and 4, they named as many professions and first names as possible, respectively. We averaged the number of answers across items (α = 83).

### Creativity.

*Divergent thinking*. We assessed divergent thinking ability with an alternative uses task similar to the one used in the eye-tracking paradigm (objects: book, car tire, tin can). However, in this test, participants typed in all their ideas they had during the 2.5 minutes time per item. Six raters (α = .80) evaluated creativity of ideas on a 4-point scale from 0 *not creative* to 3 *very creative* (Silvia et al., 2008). Top-3 creativity scores were averaged across items. Additionally, average number of ideas was used as divergent thinking fluency score.

*Creative Activities*. We assessed self-reported creative activities with an adapted short form of the Inventory of creative activities and achievements (ICAA, Diedrich et al., 2018). Participants indicated how often they were engaged in activities from various domains (literature, music, handcraft, decoration, cooking, fine arts, performing arts, technical/science, and social) within the last 12 months on a 5-point scale from 0 *never* to 4 *very often/daily*. The average score was used (α = .70).

*Self-rated Creativity*. We assessed self-perceived creativity using a single question asking “How creative are you in relation to others?” on a 10-point scale ranging from 1 *not creative at all* to 10 *very creative*.

*Creative Achievements*. We assessed creative achievements by asking participants for their three most creative achievements. Five raters evaluated creativity of each achievement on a 6-point scale from 0 *not creative* to 5 *genius* (α = .89, Diedrich, Benedek, Jauk, & Neubauer, 2015). We built an average score across achievements (using a max score yielded the same results).

### Personality.

*Openness*. We assessed openness to experience with the subscale Openness from the German version of the Big Five Inventory (BFI, Lang, Lüdtke, & Asendorpf, 2001). Participants evaluated how well 10 statements described them on a 5-point scale ranging from 1 *not at all* to 5 *very well*. Answers were averaged (α = .80).

## Results and Discussion

Descriptive statistics are given in Table S1 and correlation matrix in Table S3.

We explored which measures best predict distraction rate at by starting with a complete multiple regression model and removing measures with the lowest t-value stepwise until further removal decreased model fit significantly.

The full model including all measures was significant (*F*_16,93_ = 1.85, *p* = .036, R^2^ = .24, R^2^_adj._ = .11). The final model (Table S2) included one measure per cluster: SIPI poor attention control from the attention cluster, verbal fluency from the executive functions cluster, creative achievements from the creativity cluster and openness from the personality traits. Working memory capacity did not predict distraction rate.

Interestingly, our broad set of measures explained only 24% of variance in the distraction rate. The final model explained only 16%. The result highlights that there might be several different reasons leading one to ignore or attend to visual distractors. For example, some might have looked at more pictures because they had bad attention control, while others might have looked at more pictures because they had enough cognitive resources to look at them without interference of the main task. Similarly, some participants might have actively integrated the pictures in their creative process while others had no problem completely shutting out the visual world during their creative process. Further studies might disentangle potential causes of external attention during creative processes by administering detailed questions after each block and task.

| Table S2.1  *Descriptive Statistics of the individual differences measures (N = 144).* | | | | | | | |
| --- | --- | --- | --- | --- | --- | --- | --- |
|  | *M* | *SD* | Median | Min | Max | Skew | Kurtosis |
| Distraction rate (%) | 39.37 | 28.53 | 33.33 | 1.39 | 98.61 | 0.52 | -1.02 |
| Age | 24.12 | 4.35 | 24 | 19 | 40 | 1.04 | 1.03 |
| Operation Span | 0.74 | 0.18 | 0.79 | 0.00 | 0.95 | -1.69 | 3.77 |
| Flanker | -9.06 | 12.16 | -7.68 | -84.00 | 11.51 | -3.64 | 18.54 |
| Antisaccade | 0.31 | 0.16 | 0.30 | -0.01 | 0.71 | 0.23 | -0.53 |
| SIPI poor attention control | 47.15 | 8.92 | 46.00 | 21.00 | 71.00 | 0.17 | -0.22 |
| ASRS | 2.33 | 1.38 | 2.00 | 0.00 | 6.00 | 0.03 | -0.60 |
| Letter sets | 7.28 | 2.41 | 7.00 | 2.00 | 14.00 | 0.53 | 0.12 |
| Number series | 6.42 | 4.00 | 6.00 | 0.00 | 15.00 | 0.52 | -0.73 |
| Paper folding | 5.38 | 2.17 | 5.00 | 1.00 | 10.00 | 0.06 | -0.62 |
| Verbal fluency | 25.52 | 4.45 | 26.00 | 15.25 | 36.75 | -0.13 | -0.35 |
| DT top-3 creativity | 1.33 | 0.26 | 1.31 | 0.74 | 2.22 | 0.35 | 0.31 |
| DT fluency | 7.90 | 3.10 | 7.33 | 3.00 | 17.00 | 0.67 | -0.19 |
| Creative Activities | 1.62 | 0.63 | 1.56 | 0.33 | 3.78 | 0.71 | 0.94 |
| Creative Achievements | 1.31 | 0.52 | 1.33 | 0.07 | 2.93 | 0.09 | 0.19 |
| Self-rated Creativity | 5.92 | 2.11 | 6.00 | 1.00 | 10.00 | -0.27 | -0.90 |
| Openness | 3.85 | 0.50 | 3.80 | 2.40 | 4.90 | -0.08 | -0.42 |
| *Note*. DT = divergent thinking, ASRS = Adult ADHD Self-Resport Scale v1.1, SIPI = Short Imaginal Processes Inventory. | | | | | | | |

| Table S2.2  *Final model predicting distraction rate.* | | | | | |
| --- | --- | --- | --- | --- | --- |
|  | β | *b* | CI 95% | *t* | *p* |
| Intercept |  | 39.40 | 35.48, 44.13 | 17.66 | < .001 |
| Age | .10 | 0.66 | -0.43, 1.87 | 1.23 | .209 |
| SIPI poor attention control | .18 | 0.57 | 0.05, 1.06 | 2.20 | .029 |
| Verbal fluency | -.22 | -1.38 | -2.34, -0.38 | -2.73 | .007 |
| Creative achievements | -.16 | -7.36 | -14.83, -0.21 | -2.07 | .040 |
| Openness | .22 | 12.46 | 4.69, 20.96 | 2.71 | .008 |
|  |  |  |  |  |  |
| *R*^2^ = .16, *R*^2^_adj._ = .13 |  |  |  |  |  |
| *F*_5,136_ = 5.04, *p* < .001 |  |  |  |  |  |
| *Note*. SIPI = Short Imaginal Processes Inventory. | | | | | |

| Table S2.3  *Correlation matrix*. | | | | | | | | | | | | | | | | | |
| --- | --- | --- | --- | --- | --- | --- | --- | --- | --- | --- | --- | --- | --- | --- | --- | --- | --- |
|  |  | 1 | 2 | 3 | 4 | 5 | 6 | 7 | 8 | 9 | 10 | 11 | 12 | 13 | 14 | 15 | 16 |
| 1 | Distraction rate (%) | 1 |  |  |  |  |  |  |  |  |  |  |  |  |  |  |  |
| 2 | Age | .13 | 1 |  |  |  |  |  |  |  |  |  |  |  |  |  |  |
| 3 | Operation Span | -.07 | -.01 | 1 |  |  |  |  |  |  |  |  |  |  |  |  |  |
| 4 | Antisaccade | .13 | -.14 | -.28 | 1 |  |  |  |  |  |  |  |  |  |  |  |  |
| 5 | Flanker | -.05 | .14 | .16 | -.13 | 1 |  |  |  |  |  |  |  |  |  |  |  |
| 6 | SIPI poor attention control | .16 | .13 | -.14 | .03 | -.01 | 1 |  |  |  |  |  |  |  |  |  |  |
| 7 | ASRS | .13 | -.06 | -.04 | -.09 | -.03 | .32 | 1 |  |  |  |  |  |  |  |  |  |
| 8 | Letters sets | -.23 | -.03 | .01 | -.20 | .11 | -.17 | -.15 | 1 |  |  |  |  |  |  |  |  |
| 9 | Number series | -.04 | -.07 | -.04 | -.13 | .15 | -.01 | .02 | .50 | 1 |  |  |  |  |  |  |  |
| 10 | Paper folding | -.08 | -.14 | .02 | -.26 | .01 | -.18 | .04 | .32 | .26 | 1 |  |  |  |  |  |  |
| 11 | Verbal fluency | -.24 | -.03 | .17 | -.02 | .03 | -.10 | -.16 | .30 | .14 | .00 | 1 |  |  |  |  |  |
| 12 | DT top-3 creativity | -.10 | .00 | .03 | -.03 | .00 | -.04 | .00 | .20 | .20 | .16 | .40 | 1 |  |  |  |  |
| 13 | DT fluency | .00 | .00 | -.01 | .02 | -.05 | .01 | .09 | .06 | .02 | .06 | .21 | .43 | 1 |  |  |  |
| 14 | Creative activities | .03 | -.17 | .01 | .01 | -.09 | -.26 | .02 | .10 | -.10 | .07 | .13 | .16 | .16 | 1 |  |  |
| 15 | Self-rated creativity | .06 | .02 | -.01 | -.05 | .09 | -.22 | .03 | -.02 | .03 | .12 | .02 | .23 | .23 | .36 | 1 |  |
| 16 | Creative achievements | -.15 | .02 | -.01 | -.07 | .01 | .01 | -.05 | .08 | .12 | .10 | .05 | .14 | -.15 | -.05 | .10 | 1 |
| 17 | Openness | .17 | .04 | -.09 | .04 | -.04 | -.20 | .12 | -.07 | -.04 | .02 | .03 | .16 | .22 | .44 | .62 | .11 |
| *Note*. DT = divergent thinking, ASRS = Adult ADHD Self-Resport Scale v1.1, SIPI = Short Imaginal Processes Inventory. | | | | | | | | | | | | | | | | | |

# References

Amthauer, R., Brocke, B., Liepmann, D., & Beauducel, A. (2001). *Intelligenz-Struktur-Test 2000 R*. Göttingen: Hogrefe. https://doi.org/10.1026//1617-6391.1.1.50

Diedrich, J., Benedek, M., Jauk, E., & Neubauer, A. C. (2015). Are Creative Ideas Novel and Useful? *Psychology of Aesthetics, Creativity, and the Arts*, *9*(1), 35–40. https://doi.org/10.1037/a0038688

Diedrich, J., Jauk, E., Silvia, P. J., Gredlein, J. M., Neubauer, A. C., & Benedek, M. (2018). Assessment of real-life creativity: The inventory of creative activities and achievements (ICAA). *Psychology of Aesthetics, Creativity, and the Arts*, *12*(3), 304–316. https://doi.org/10.1037/aca0000137

Eichele, T., Debener, S., Calhoun, V. D., Specht, K., Engel, A. K., Hugdahl, K., … Ullsperger, M. (2008). Prediction of human errors by maladaptive changes in event-related brain networks. *Proceedings of the National Academy of Sciences of the United States of America*, *105*(16), 6173–8. https://doi.org/10.1073/pnas.0708965105

Ekstrom, R. B., French, J. W., Harman, H. H., & Derman, D. (1976). *Manual for Kit of factor-referenced cognitive tests*. Princeton, NJ: Educational Testing Service.

Huba, G. J., Aneshensel, C. S., & Singer, J. L. (1981). Development of scales for three second-order factors of inner experience. *Multivariate Behavior Research*, (16), 181–206. https://doi.org/10.1207/s15327906mbr1602_4

Humphreys, M. S., & Revelle, W. (1984). Personality, Motivation, and Performance: A Theory of the Relationship Between Individual Differences and Information Processing. *Psychological Review*, *91*(2), 153–184. https://doi.org/10.1037/0033-295X.91.2.153

Jangraw, D. (2015). PsychoPy Implementation of the Erikson Flanker Task described in Eichele 2008. Retrieved from https://github.com/djangraw/PsychoPyParadigms

Kane, M. J., Bleckley, M. K., Conway, A. R., & Engle, R. W. (2001). A controlled-attention view of working-memory capacity. *Journal of Experimental Psychology. General*, *130*(2), 169–183. https://doi.org/10.1037/0096-3445.130.2.169

Lang, F., Lüdtke, O., & Asendorpf, J. (2001). Testgüte und psychometrische Äquivalenz der deutschen Version des Big Five Inventory (BFI) bei jungen, mittelalten und alten Erwachsenen. *Diagnostica*, *47*(3), 111–121. https://doi.org/10.1026//0012-1924.47.3.111

Silvia, P. J., Beaty, R. E., & Nusbaum, E. C. (2013). Verbal fluency and creativity: General and specific contributions of broad retrieval ability (Gr) factors to divergent thinking. *Intelligence*, *41*(5), 328–340. https://doi.org/10.1016/j.intell.2013.05.004

Silvia, P. J., Winterstein, B. P., Willse, J. T., Barona, C. M., Cram, J. T., Hess, K. I., … Richard, C. A. (2008). Assessing creativity with divergent thinking tasks: Exploring the reliability and validity of new subjective scoring methods. *Psychology of Aesthetics, Creativity, and the Arts*, *2*(2), 68–85. https://doi.org/10.1037/1931-3896.2.2.68

von der Malsburg, T. (2015). Py-Span-Task -- A software for testing working memory span. https://doi.org/10.5281/zenodo.18238

World Health Organization. (2004). *Screening-Test mit Selbstbeurteilungs-Skala V1.1 für Erwachsene mit ADHS (ASRS-V1.1)*. Retrieved from https://www.hcp.med.harvard.edu/ncs/ftpdir/adhd/6Q_German_final.pdf
