# Supplementary material for: Eye behavior predicts susceptibility to visual distraction during internally directed cognition
Source: Atten Percept Psychophys. 2020 Jun 4;82(7):3432–44. doi: 10.3758/s13414-020-02068-1 (PMC7536161; doi:10.3758/s13414-020-02068-1)
Supplement: Supplementary file 3 — (DOCX 376 kb) [file 13414_2020_2068_MOESM4_ESM.docx]

**Supplemental material 3 of manuscript:**

**Eye behavior predicts susceptibility to visual distraction during internally directed cognition**


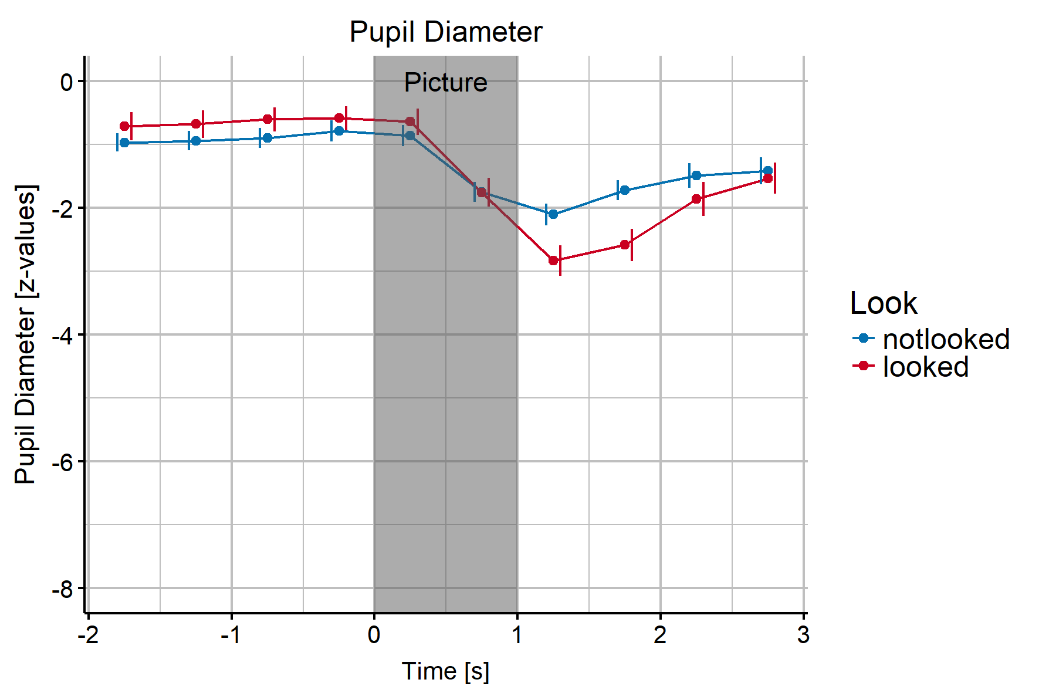
Graphical analysis of the time course of eye parameters relative to distractor onset separately for trials with and without visual distraction. Data are binned for 0.5-s intervals so they also work for discrete measures like blinks and saccades.

*Figure S3.1.* Time course of pupil dilation relative to distractor onset for trials without and with visual distraction (i.e., not looked vs looked).


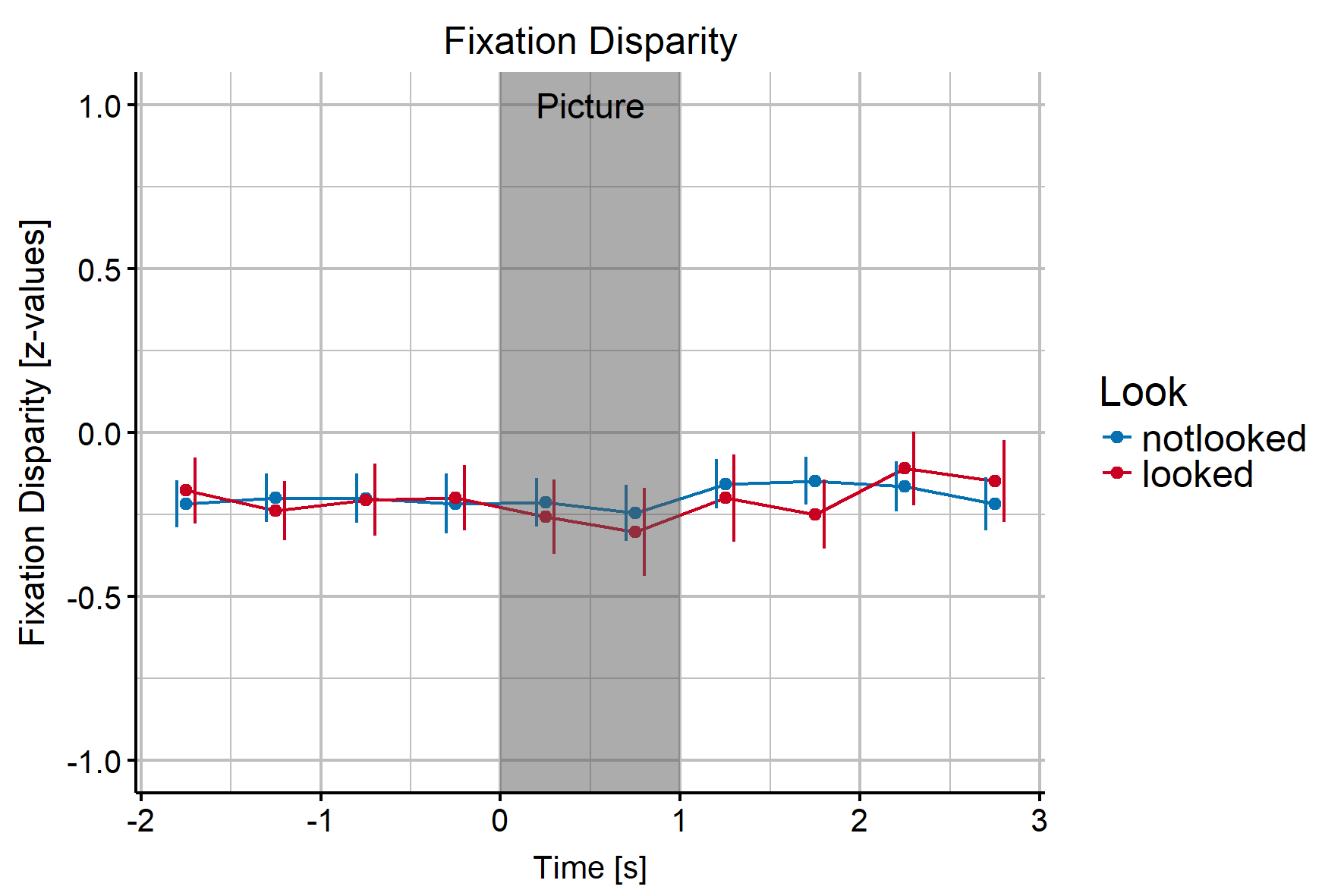


*Figure S3.2.* Time course of fixation disparity relative to distractor onset for trials without and with visual distraction (i.e., not looked vs looked).


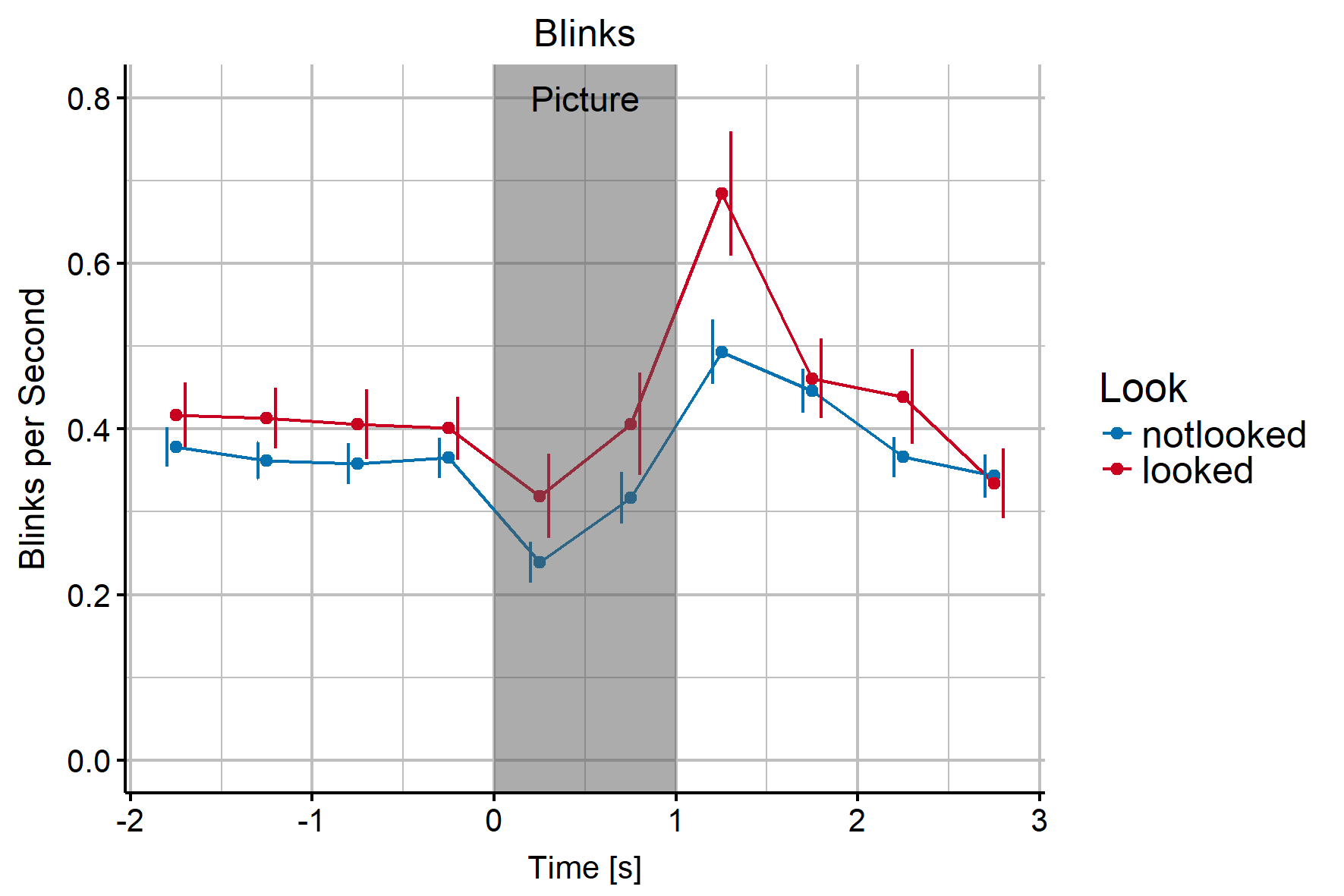


*Figure S3.3*. Time course of blink rate relative to distractor onset for trials without and with visual distraction (i.e., not looked vs looked).


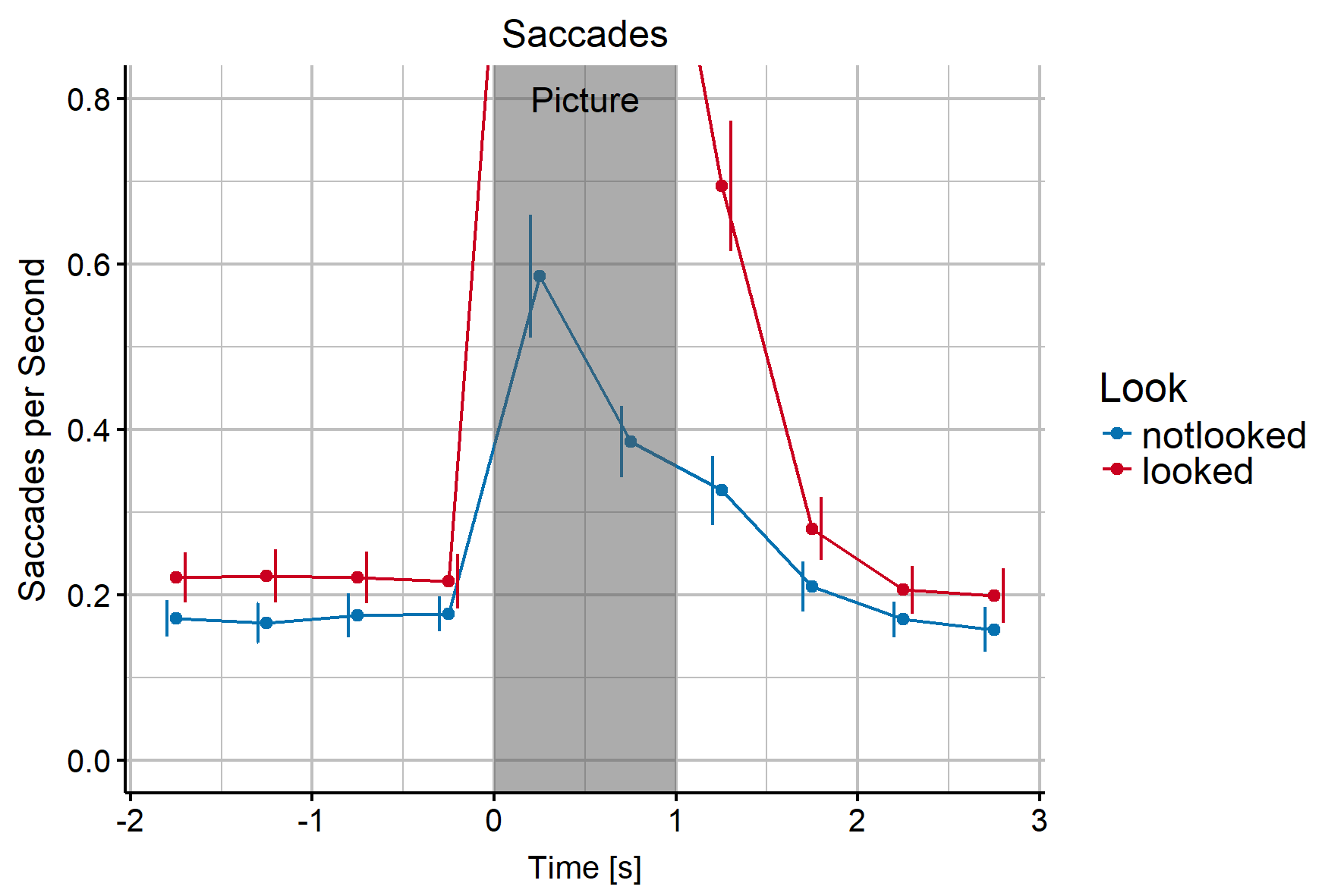


*Figure S3.4*. Time course of saccade rate relative to distractor onset for trials without and with visual distraction (i.e., not looked vs looked).
